# Modeling the effect of scale deposition on heat transfer in injection molding

**DOI:** 10.1038/s41598-025-98657-x

**Published:** 2025-04-22

**Authors:** Béla Zink, Anna Héri-Szuchács, Szabolcs Hajagos, József Gábor Kovács

**Affiliations:** 1 Department of Polymer Engineering, Faculty of Mechanical Engineering, Budapest University of Technology and Economics, Műegyetem rkp. 3, Budapest, 1111 Hungary; 2 MTA-BME Lendület Lightweight Polymer Composites Research Group, Műegyetem rkp. 3, Budapest, 1111 Hungary

**Keywords:** Additive manufacturing, Conformal cooling channels, Injection molding, Scale deposition, Heat transfer efficiency, Finite element modeling, Mechanical engineering, Materials science

## Abstract

The advent of additive manufacturing technologies has revolutionized the design and application of injection molds with conformal cooling systems. These mold inserts, manufactured directly from metal powder, feature complex geometries that align with the part contours, enabling more efficient and uniform heat extraction than traditional cooling methods. However, over time, even the most efficient cooling circuits degrade due to corrosion, limescale, and other deposition on the cooling channel walls, significantly decreasing heat transfer. We developed finite element models to simulate various deposition scenarios to address the complexity of modeling these depositions and their impact on heat transfer. Our study evaluates the thermal conductivity and thickness of deposit layers in conventional and conformal cooling channels. The resulting universal model accurately predicts the cooling efficiency of injection molds produced of various mold material, considering arbitrary deposition characteristics, e.g. limescale and corrosion. This model makes optimizing cooling systems for injection molds easier, thus providing crucial information on the time limit of the predictive maintenance and the mold performance change.

## Introduction

Injection molding is one of the most widely used plastic processing technologies, which accounts for more than 30% of the polymer products market^1^. Injection molding offers fast and economically efficient production of plastic parts of various shapes and complex geometries. The cost-effectiveness and quality of injection-molded parts highly depend on the cooling step in the process, which can take up to 80% of the overall cycle time^2^. One way to significantly improve the cooling efficiency of injection molds is to use conformal cooling channels (CCCs), which are placed equidistant from the cavity surfaces. With CCCs, cooling time can be reduced by 30–50% compared to conventional straight-drilled cooling channels^3,4^. Moreover, CCCs assure more uniform heat removal^5^, which minimizes the warpage and shrinkage of injection-molded parts. Another approach to enhance the cooling efficiency of an injection mold is to make it partially or entirely from materials with high thermal conductivity, such as copper, beryllium-copper^6^, and aluminum alloys^7^. In many cases, both CCCs and high thermal conductivity materials are used to increase the cooling efficiency of the mold. For example, Torres-Alba et al.^8^ used CCCs in combination with a mold insert manufactured from Fastcool^®^ material, which is steel with high thermal conductivity (~ 50 W/m∙K). With such a hybrid design, the authors reduced the cycle time for a complex-shaped part by 27%. Ahn and Kim^9^ used CCCs and three layers of materials in one mold: P21 tool steel in the cavity area for high strength, Ampcoloy 940 in the bottom part to dissipate heat, and a special nickel-copper alloy as a mid-layer to decrease thermal stresses. This allowed the authors to reduce the cooling time from 15 to 3 s. Imran et al.^10^ produced a die for high-pressure die casting made from copper with a 2 mm layer of H13 tool steel deposited on it. The proposed solution reduced solidification time by up to 35% compared to conventional steel dies.

Although CCCs and material choice can significantly improve heat extraction in injection molds, cooling efficiency can still be impaired during mold operation due to fouling (unwanted material deposits) inside cooling channels. Fouling can occur due to corrosion, the deposition of biological cells, chemical reactions, crystallization, and other reasons^11^. However, crystallization fouling (also called “scaling”) is the most relevant type of fouling for cooling channels^12^. Scaling is caused by inorganic salts in the working fluid, mostly calcium carbonate or calcium sulfate (CaCO_3_ and CaSO_4_)^13^. Both salts exhibit inverse solubility, which means that the solution of CaCO_3_ and CaSO_4_ in water becomes supersaturated as temperature rises, which causes the gradual deposition of dissolved salts on a heat transfer surface in the form of crystals.

**Table 1:** Thermal conductivities of different materials.

Material	Crystal form	Thermal conductivity k, W/(m∙K)	References
CaCO_3_	Calcite	0.93	^28^
	Aragonite	2.00	^29^
	Vaterite	N/A	–
CaSO_4_	Gypsum (CaSO_4_∙2H_2_O)	1.25	^29^
	Calcium sulfate hemihydrate (CaSO_4_⋅$\:\frac{1}{2}$H_2_O)	0.74	^28^
	Anhydrite (CaSO_4_)	5.80	^29^
Biofilm		0.60	^28^
Aluminum		230	–
Copper		386	^30^
Stainless steel		25	
Maraging steel (MS1)		14.20–15.80	

When CaCO_3_ crystallizes from a solution, it appears in three forms: calcite, aragonite, and vaterite (Table 1), with rhombohedral, needle-like, and hexagonal structures, respectively^14^. Calcite is the most thermodynamically stable form, followed by aragonite and vaterite^15^. At room temperature, vaterite is the dominant crystal form, while at a temperature higher than 50 °C, aragonite becomes the dominant product. CaSO_4_ also appears in three crystal forms, namely, gypsum (CaSO_4_·2H_2_O), calcium sulfate semihydrate (CaSO_4_·0.5H_2_O), and anhydrite (CaSO_4_). Gypsum is the most common crystal form, appearing in the temperature range from 40 °C to 98 °C. CaSO_4_·0.5H_2_O is generally a metastable crystal form and is much less abundant. Anhydrite gradually replaces gypsum at temperatures higher than 50 °C. Scaling exhibits considerably different thermal properties than the heat transfer surface. For example, aragonite and gypsum have a thermal conductivity of ~ 2.00 and 1.25 W/(m K), respectively, while those of steel and aluminum are ~ 15 and 230 W/(m∙K), respectively. As a result, scaling in the cooling channels can considerably decrease heat removal efficiency in an injection mold.

Scaling on the heat transfer surface is a complex process influenced by fluid velocity, flow regime, working fluid temperature, heat exchanger surface’s nature, heat transfer mode, and water composition^13^. Kim and Cho^16^ recorded a CaCO_3_ fouling process on a heat transfer surface with video, where artificially hardened water at 23 °C moved at a velocity of 0.37 m/s. They concluded that temperature, flow velocity, and water hardness affect the crystal growth behavior of CaCO_3_. Also, they proved an asymptotic growth of crystal fouling. Chen et al.^17^ studied the crystallization fouling of CaSO_4_ on heat exchanger surfaces of four different metals: copper, aluminum, brass, and stainless steel. They found that the amount of fouling increases with the thermal conductivity of the metal (stainless steel < brass < aluminum < copper). Teng et al.^18^ proved a similar tendency for CaCO_3_ deposition. They showed that CaCO_3_ deposition increases linearly with the thermal conductivity of a heat transfer surface. Hatte et al.^13^ experimentally investigated CaCO_3_ and CaSO_4_ deposition in copper tubes with modified inner surface wettability. The cooling water with various fouling salt concentrations circulated in cooling channels at different flow rates. They found that fouling resistance decreases if the Reynolds number (Re) increases and the threshold time for critical fouling increases.

Several studies focus on the analytical and numerical modeling of fouling on heat transfer surfaces in heat exchangers. For example, Quan et al.^19^ developed an analytical heat and mass transfer model to predict the fouling of CaCO_3_ on a heat transfer surface. Their model is based on the Kern-Seaton model^20^, which considers crystallization and particle fouling and predicts fouling deposition and removal rates. The modeled results showed less than a 15% deviation from the experimental data. Paakkonen et al.^21^ combined Computational Fluid Dynamics (CFD) and crystallization fouling modeling to predict the mass deposition rate of CaCO_3_ on the heated surface of a heat exchanger. They showed that the two most crucial parameters affecting crystallization fouling are surface temperature and shear stress. However, the influence of surface temperature is almost 30 times greater than the effect of shear stress.

Although the modeling of scale deposition is quite well developed for heat exchanger surfaces, this problem has rarely been studied for injection molding cooling channels. To our knowledge, only three articles have been published recently on this topic^7,22,23^. The effect of limescale and rust in injection molding was analyzed by Novoplan GmbH^22^ using the Moldex 3D injection molding simulation software. The authors concluded that in the case of their box-shaped product, 1 mm of rust increases mold surface temperature by 20 °C and warpage by 0.4 mm. 1 mm of limescale doubles those values caused by rust. Furthermore, the efficiency of the cooling circuits drops by 6% in the case of 1 mm of limescale. The authors did not specify the thermal conductivity of the depositions or whether the thermal conductivities were measured or just assumed. Zink and Kovacs^7^ developed a numerical model to investigate the effect of limescale that formed on the wall of the conformal cooling channels in a mold insert made from maraging steel MS1 and Ampcoloy 88. They measured the thermal properties of limescale and found that its average thermal conductivity was 1.37 W/(m K). The authors also concluded that a 2 mm thick limescale hinders the heat extraction of CCCs and reduces it to the heat extraction of straight-drilled cooling channels. Poszwa and Szostak^23^ conducted numerical simulations to estimate the impact of the thickness of a fouling layer on the distribution of temperature on the mold cavity surface and on solidification time. They found that a scale deposition layer of 0.25 mm does not considerably extend the solidification time. However, a scale deposition layer of 1 mm significantly decreases the efficiency of cooling channels and increases solidification time.

Conformal cooling channels are generally more prone to corrosion than conventional cooling channels because the Direct Metal Laser Sintering (DMLS) and Electron Beam Melting technologies result in higher surface roughness in the internal surfaces like the cooling channels. Higher surface roughness can accumulate corrosion agents, like salts. Furthermore, higher surface roughness can disrupt the formation of oxide layers^24^.

The current study aims to establish a universal model that can calculate the efficiency of conformal and conventional cooling channels affected by scale deposition. The model calculates efficiency as a function of the heat conductivity of the scale deposition and deposition thickness. This study also examines the influence of scale deposition on the thermal conductivity of two types of molds: those produced from steel and those from a copper alloy.

## Materials and methods

The numerical experiments aimed to investigate the effect of deposits forming in the cooling channels. We established an analytical model using the results of numerical modeling to calculate the effect of deposition thickness and deposition conductivity on the cooling efficiency of different mold designs and mold materials.

### Mold materials

We used different mold materials in the calculations and developed the model. MaragingSteel MS1 (MS1, EOS GmbH, Germany, Gräfelfing) is a general steel material used to manufacture injection molds. Ampcoloy 88 (AMP, Ampco Metal S.A., Switzerland, Marly) is a highly alloyed copper material; it is mainly used to manufacture special injection mold inserts, which enhance cooling efficiency locally. These two mold materials represent very different thermal conductivities: 20 W/(mK) and 230 W/(mK) (Table 2) and cover the thermal conductivity spectrum of materials used for manufacturing high-series injection molds.

**Table 2:** The main characteristics of the materials used in the simulations.

Property	MS1	Ampcoloy 88
Density (kg/m^3^)	8100	8750
Thermal conductivity coefficient (W/(m K))	20	230
Specific heat capacity (J/(kg K))	450	420
Young’s modulus (GPa)	205	130

### Mold design

**Fig. 1:**
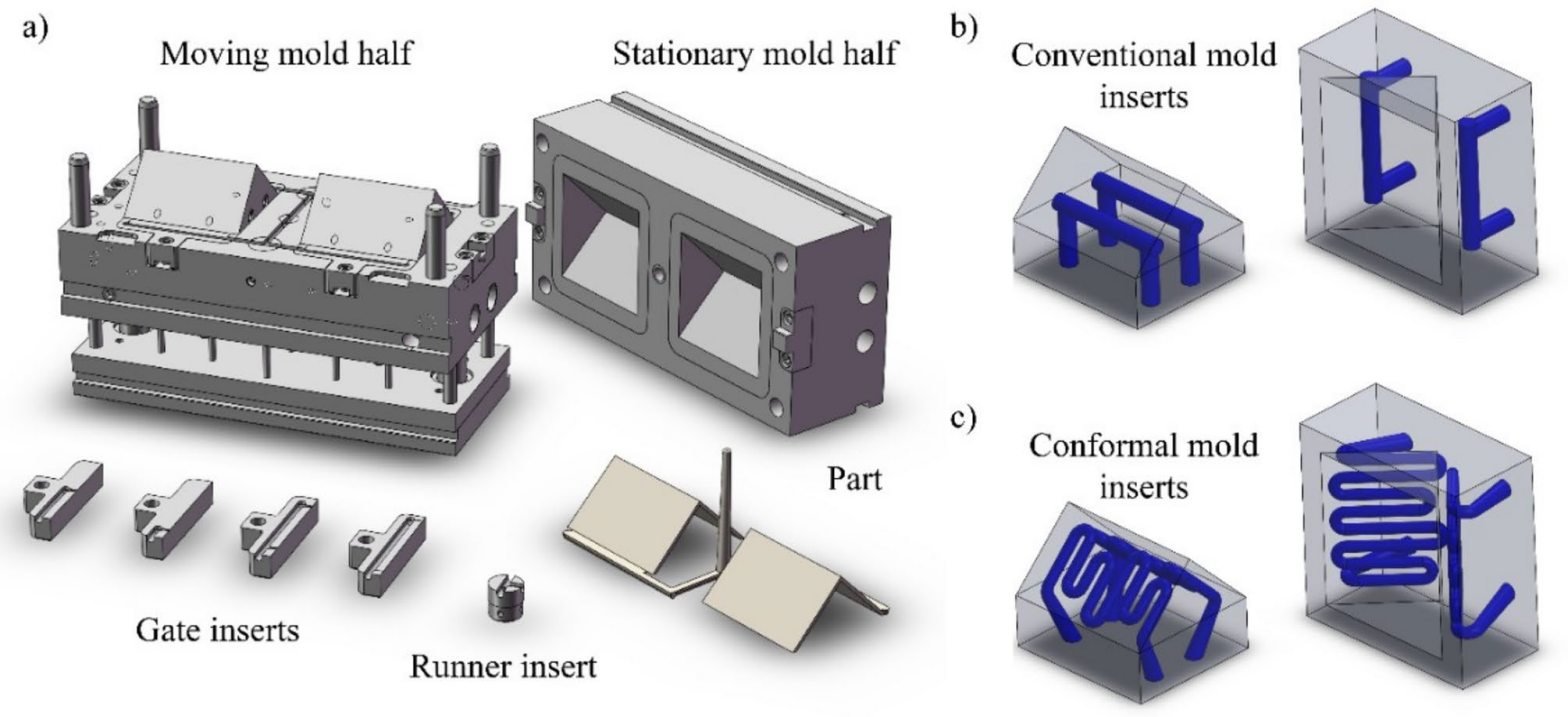
The injection mold block used in the simulations (**a**) the Conventional (**b**) and the Conformal (**c**) inserts with highlighted cooling systems.

We used a two-cavity injection mold to model depositions. It was, however, simplified to a single-cavity mold to eliminate the cross effects. The mold can be used with pinpoint and film gate inserts. In the case of the pinpoint gates, single-gate and double-gate designs exist. For modeling, we used the film gate insert. Two different cooling layouts (CL) were modeled in a single-cavity mold (Fig. 1a) for the cooling simulations: a conventional (Fig. 1b) (*Conventional AMP and Conventional MS1 inserts*) and a conformal (Fig. 1c) (*Conformal AMP and Conformal MS1 inserts*) CL. Cooling channel size in the case of conventional channels was set to 8 mm because the thickness of the part was 2 mm. The distance between the surface and the centerline of the cooling channel was 13.2 mm for the core, and 12 mm for the cavity insert. The conformal cooling channels had smaller diameter and depth; however, heat removal was uniform because the cooling channels followed the geometry of the part. The conformal layout had a cooling channel diameter of 5 mm, the channel depth was 6.5 mm, and the distance between the two channels was 8.5 mm.

### Deposition parameters

The specific heat and the density of the deposition were set to 800 J/(kg °C) and 1.2 g/cm^3^, respectively^7,27^. We varied the thermal conductivity of the deposition between 0 W/(m K) and the thermal conductivity of the mold inserts (20 and 230 W/(m K)). For the steel inserts, the following thermal conductivities were used: 0.01, 0.05, 0.1, 0.25, 0.5, 1, 2, 5 and 20 W/(mK), while for the copper inserts, 0.01, 0.05, 0.1, 0.25, 0.5, 1, 2, 5 and 230 W/(mK).

### Governing equations and boundary conditions

The temperature field was calculated using cycle-averaged calculations. We made the following assumptions for the calculations^26^:

- Mold, coolant, and material properties, such as thermal and physical properties, are constant.
- The melt and the mold contact is assumed to be perfect, without air gaps. The interfacial temperature distribution is the same for both materials.
- The coolant flow rate is assumed to be sufficiently large; therefore, the coolant temperature is constant in the calculations.

Laplace’s equation governing the cycle-averaged calculations^26^:

1 $$\:{\nabla\:}^{2}T=0.$$

The boundary conditions for each surface are^26^:

On the mold cavity surface

2 $$\:{{\text{k}}_{mold}\frac{\partial\:T}{\partial\:n}}^{2}=\stackrel{-}{q}$$

On the cooling channel surface

3 $$\:{\text{k}}_{mold}\frac{\partial\:T}{\partial\:n}=-{h}_{mold-coolant}(T-{T}_{c})$$

On the mold exterior surface

4 $$\:{\text{k}}_{mold}\frac{\partial\:T}{\partial\:n}=-{h}_{mold-air}(T-{T}_{a})$$

**Fig. 2:**
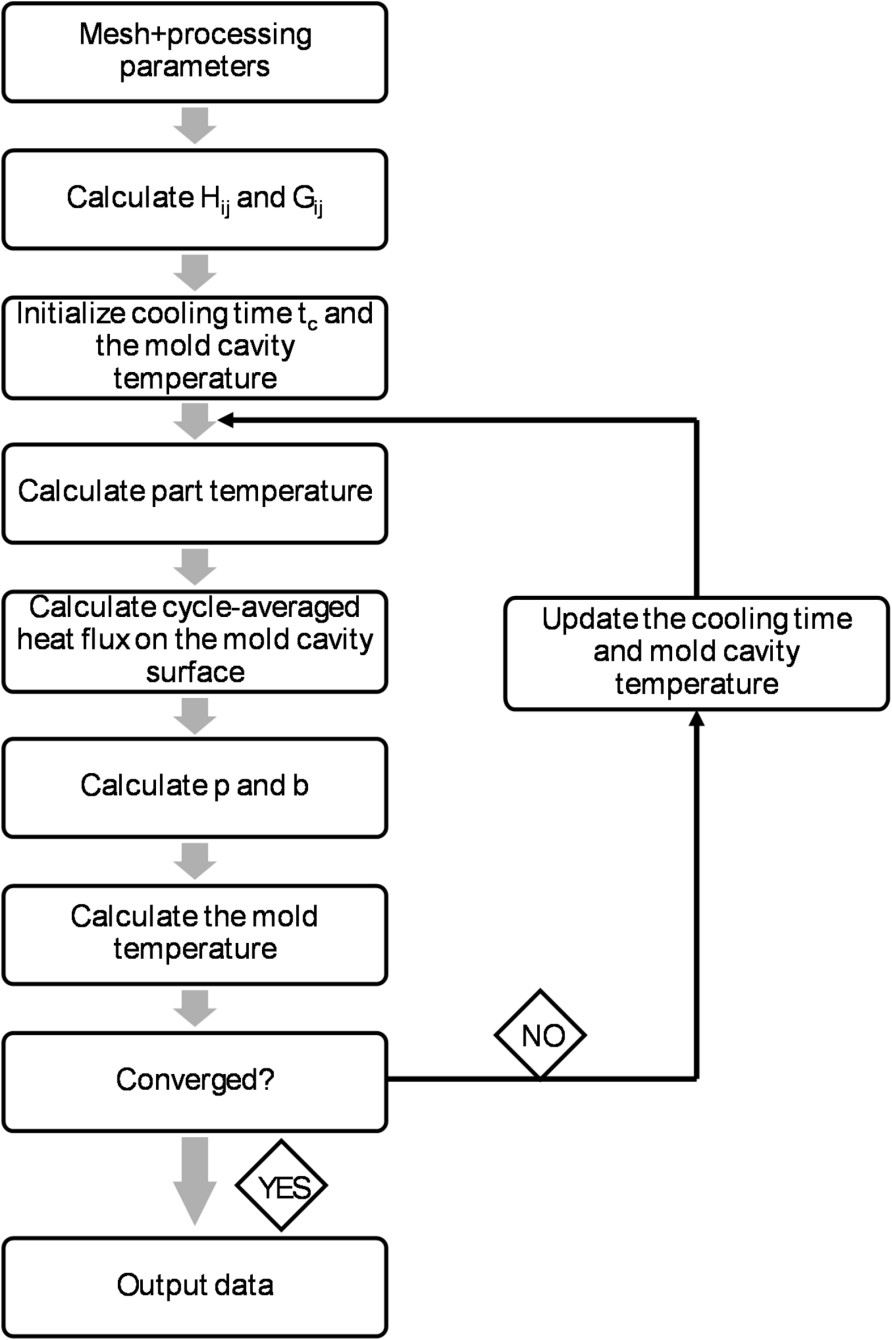
Flowchart of cycle-averaged cooling calculations^26^.

The first step of the solution of Laplace’s equation (Fig. 2) is to calculate the part temperature and cycle-averaged heat flux using the boundary element model. For the calculations (Eqs. (5)–(10)), the initial mold cavity temperature and cooling time are used. Initial cooling time is estimated based on the processing parameters, part geometry and material^26^.

5 $$\:{\widehat{H}}_{ij}={\int\:}_{{\varDelta\:}_{j}}^{}{q}^{*}\left(y,\:x\right)ds={\int\:}_{{\varDelta\:}_{j}}^{}\frac{\partial\:{T}^{*}(y,\:x)}{\partial\:n}ds$$

6 $$\:{H}_{ij}=\left\{\begin{array}{c}{\widehat{H}}_{ij}\:when\:i\ne\:j\\\:{\widehat{H}}_{ij}+\frac{1}{2}\:\:when\:i=j\end{array}\right.$$

7 $$\:{G}_{ij}={\int\:}_{{\varDelta\:}_{j}}^{}{T}^{*}\left(y,\:x\right)ds={\int\:}_{{\varDelta\:}_{j}}^{}\frac{1}{4\pi\:r(y,x)}ds$$

8 $$\:\frac{\partial\:T}{\partial\:t}=a\frac{\partial\:T}{\partial\:{s}^{2}}$$

9 $$\:a=\frac{{k}_{e}}{\rho\:c}$$

10 $$\:\overline{q}=\frac{{\int\:}_{0}^{tc}q\left(t\right)dt}{tc}$$

The next step is to calculate the mold temperature (Eqs. (11)-(13)) and check if the iteration calculations converged. If the calculation converges according to the set convergence limit, the results are saved^26^.

11 $$\:{p}_{j}=\left\{\begin{array}{c}{f}_{c}\left(-{T}_{c}\right)\:\:on\:the\:cooling\:system\:surface\\\:{f}_{a}\left(-{T}_{a}\right)\:\:on\:the\:mold\:exterior\:surface\\\:-\frac{\overline{q}}{{k}_{mold}}\:\:on\:the\:mold\:cavity\:surface\end{array}\right.$$

12 $$\:\varvec{b}=\varvec{G}\varvec{p}$$

13 $$\:\stackrel{\sim}{\varvec{H}}\varvec{T}=\varvec{b}$$

The layer of deposition in the cooling channels inhibits heat removal between the mold insert and the coolant. Therefore, heat resistance consists of three elements:

- the resistivity caused by the lower heat conductivity of the deposition,
- the contact resistance between mold and deposition, and.
- the contact resistance of deposition and coolant.

The resulting heat resistivity is calculated by summarizing each element. Equation (14) describes the heat resistance caused by the deposition for a simple case where the cooling channels have a circular cross-section^27^:

14 $$\begin{aligned}R&={R}_{h,\:coolant-deposition}+{R}_{k}+{R}_{h,\:mold-deposition}\nonumber\\&=\frac{1}{2\cdot \:{r}_{1}\pi\:\cdot \:{h}_{coolant-deposition}}+\frac{ln\left(\frac{{r}_{2}}{{r}_{1}}\right)}{2\cdot \:L\cdot \:\pi\:{\cdot \:k}_{deposition}}\nonumber\\ &\quad +\:\frac{1}{2\cdot \:{r}_{2}\pi\:\cdot \:{h}_{mold-deposition}}.\end{aligned}$$

Heat transfer between the coolant and the deposition is considered to be perfect. In our calculations, we only considered the resistance caused by the low conductivity of the depositions formed in the cooling channels because we assumed this resistance was decisive.

### Numerical modeling method

**Fig. 3:**
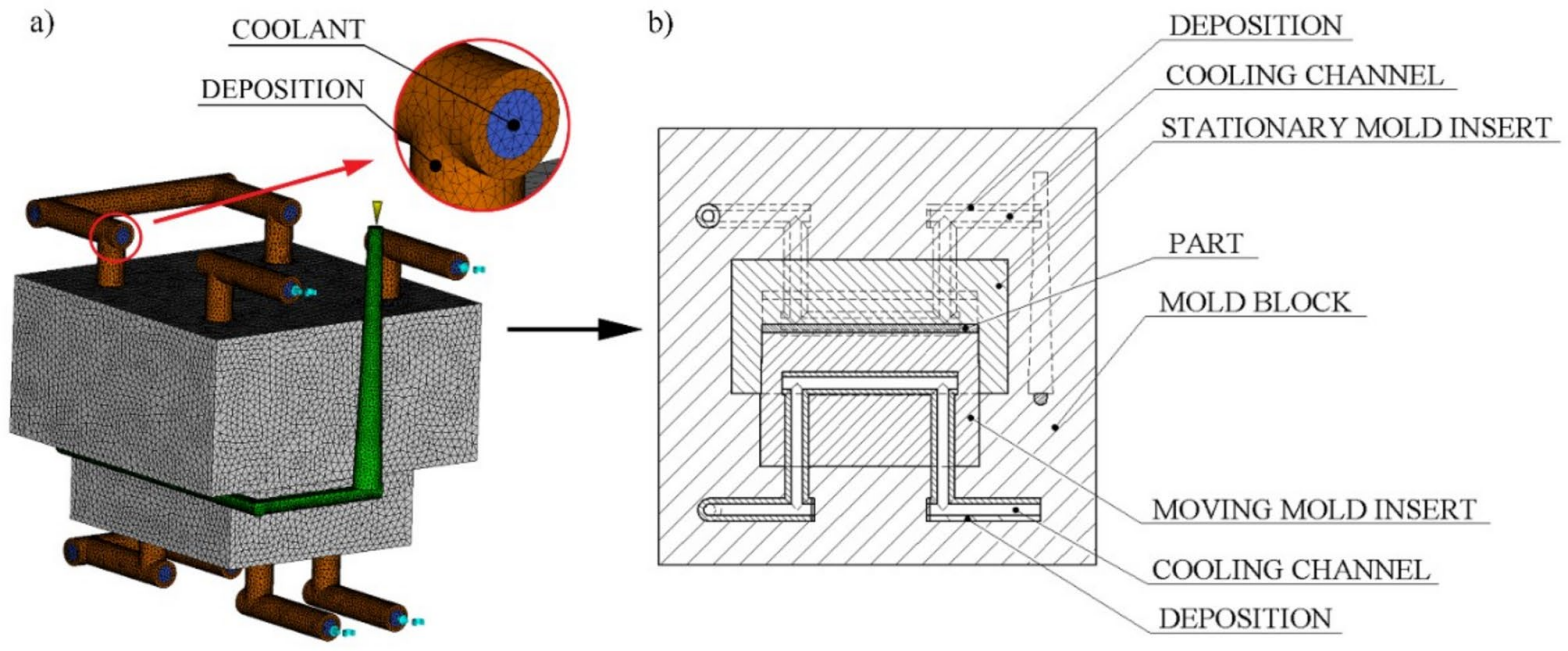
Simulation model, with the deposition (**a**) and detailed section view of the simulation model (**b**).

The simulations were carried out with Autodesk Simulation Moldflow Insight 2021, with PA (BASF, Ultramid KR 4450). Four-node tetrahedral elements were used in the entire model, including parts, mold inserts, and cooling circuits (Fig. 3.). Four-node tetrahedral elements make it possible to consider heat conduction in all directions and calculate results in all nodes. The mathematical model.

- uses 3D Navier-Stokes equations,
- calculates temperature, pressure, and three-directional velocity components at all nodes,
- considers conduction in all three directions,
- considers gravity and inertia.

We modeled the deposition into the CAD model (Solidworks 2020, Dassault Systemes, France, Vélizy-Villacoublay) and meshed the entire model in Moldflow. We examined 0.25 mm, 0.5 mm, 1 mm, and 2 mm thick depositions. We ran simulations with different mesh sizes (9.3, 5.5, 4.2, 3.25, 2.5, and 1.5 mm) to determine the right mesh size, which ensures that the results do not depend on mesh size. According to the suggestions of Autodesk Moldflow, the mesh was acceptable under 4.2 mm, as the deviation of the results was within 5%. The optimum accuracy and run time was achieved with a mesh size of 2.5 mm. Global mesh size was set to 2.5 mm, and the chord angle was set to 40°, so mesh size decreased in areas where the complex geometries, such as the gate and the cooling channels, required it. Ten layers of elements were used in the thickness direction for every component. Depending on deposition thickness, the element number was between 3.5 and 10.1 million for conformal and between 1.5 and 3.4 million for conventional cooling. To determine cooling time, we used transient thermal simulation. Cooling efficiency was calculated based on the cycle-averaged Cool FEM solver results with the conduction solver. Autodesk CFD module was used for CFD calculations. The K-epsilon turbulence model was used for the coolant flow. Part heat flux calculations were performed using Conduction solver. The Modified Petrov-Galerkin (ADV 5) advection scheme was used for energy, velocity, and pressure equations. Perfect clamping was assumed; therefore, the thermal conductance of the mold block was set to the default 30,000 W/m^2^K); all relevant parameters are listed in Table 3. Coolant inlet points were set to the near side of the gate. The coolant flow rate was set to ensure turbulent flow. We set the injection molding parameters based on injection molding trials, where cycle optimization was the goal.

**Table 3:** The parameters used in the simulation study.

Melt temperature (°C)	300
Ejection temperature (°C)	113
Ambient temperature (°C)	25
Mold surface temperature (°C)	80
Injection, packing, and cooling time together (s)	19
Mold open time (s)	6.3
Initial mold temperature (°C)	80
Coolant temperature (°C)	80
Coolant flow rate, l/min	1
Mold block conductance (W/(m^2^K))	30,000
Cool FEM transient within cycle solver parameters	
Conformal cooling solver	FEM solver
Time steps (–)	100
Mold temperature convergence tolerance (°C)	0.1
Maximum number of mold temperature iterations	100
Cool FEM averaged within cycle solver parameters	
Conformal cooling solver	FEM solver
Time steps (–)	13
Mold temperature convergence tolerance (°C)	0.1
Maximum number of mold temperature iterations	100

### Modeling method of the effect of deposition

The cooling efficiency of the injection molds varies from 0 to 100%. We assumed that the increase of cooling efficiency follows the sigmoid saturation character. We used the Hill function (Eq. (15)) to approximate the cooling efficiency of injection molds with conventional and conformal cooling systems.

15 $$\:y\left(x\right)={y}_{max}\frac{{x}^{n}}{{x}^{n}+{k}^{n}},$$

where y(x) is the end product of the Hill function, y_max_ is the saturation level, k is the positioning parameter, and n is the parameter influencing the increase of the function. An increase in y_max_ implies an increase in cooling efficiency. If parameter k increases, the curve shifts right (in the direction of larger x values). An increase in parameter n means that the saturation rate increases and the curve reaches the saturation level at a lower value.

## Results and discussion

We calculated the mold and melt temperatures for 13 timesteps to determine the cooling time needed to cool the whole part (at least 99% of the volume of the part) below ejection temperature (160 °C) (Table 4). There is no significant difference between the cooling times of the conventional and conformal AMP inserts. The minimal discrepancy can be attributed to the high thermal conductivity of the Ampcoloy inserts. A greater amount of heat is conducted to other parts of the mold, thereby diminishing the relative contribution of heat removal via the cooling channels. However, the Conformal MS1 insert has an almost 12% higher cooling time than the Conformal AMP insert. Conventional MS1 has the longest cooling time, which is 30% longer compared to the best solution.

**Table 4:** Cooling times based on the transient simulation results.

	Conventional		Conformal	
	MS1	AMP	MS1	AMP
Cooling time (s)	10	7.8	8.6	7.7
Increase in proportion to conformal AMP insert (%)	29.9	1.3	11.7	–

The study evaluated pressure, velocity, and temperature for both conformal and conventional cooling systems. The coolant temperature remained unaffected by deposit thickness, with maximum values below 90 °C in all scenarios. In the conventional system, temperature increases were observed in areas of stagnant flow.

Coolant velocity increased with greater deposit thickness in both systems, with maximum velocities reaching up to four times those observed in cases without deposition. In all instances, the Reynolds number exceeded 4000, indicating turbulent flow regimes. In scenarios involving 2 mm deposition, both conventional and conformal systems experience significant pressure increases. Notably, in the conformal system, the maximum pressure reaches 10 MPa. This pressure level exceeds the capabilities of standard commercial temperature control units, rendering it impractical for real-world applications. Consequently, such high-pressure scenarios are considered theoretical and are primarily utilized for modeling and simulation purposes.

**Fig. 4:**
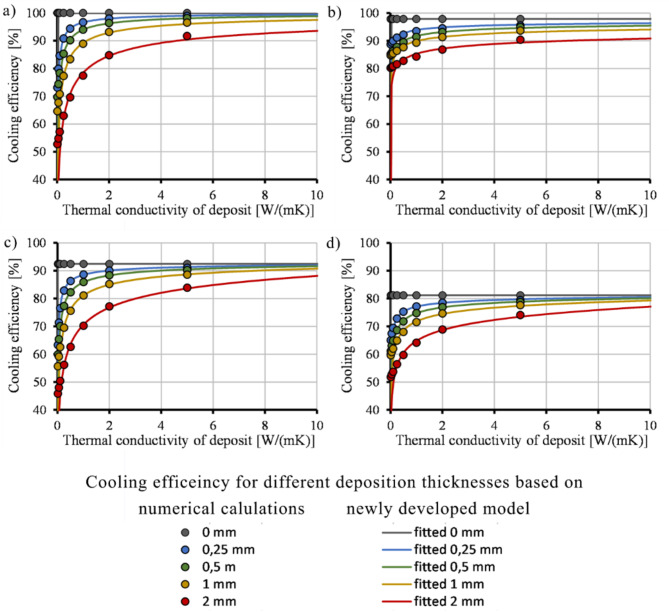
Cooling efficiency of conformal AMP (**a**), conventional AMP (**b**), conformal MS1 (**c**) and conventional MS1 (**d**) inserts as a function of deposit thickness and the fitted function on the calculated points.

We calculated relative cooling efficiency based on the heat removed (given in kW) by the cooling channels, which Moldflow calculates. The heat was used to calculate heat energy for each CL by multiplying the removed heat by the cycle time of each CL. Energy dissipated by each simulation setup was divided by the energy taken out with the most efficient construction, which is the *Conformal AMP* setup without deposition (Eq. (16)):

16 $$\:{\eta\:}_{cooling}=\frac{{\dot{Q}}_{\:channel}}{{\dot{Q}}_{.channel,\:max}}\cdot \:100,$$

where *η*_*cooling*_ (%) is the relative cooling efficiency, $\:{\dot{Q}}_{channel}$ is the heat energy removed by the cooling channels,$\:\:{\dot{Q}}_{channel,\:max}$ is the maximum heat energy removed by the cooling channels of the investigated cooling solutions. $\:{\dot{Q}}_{channel,\:max}$ and $\:{\dot{Q}}_{channel}$ are calculated based on Moldflow results. Therefore, the results show relative cooling efficiency in proportion to the best cooling setup. Theoretical cooling efficiency is 0% if the thermal conductivity of the deposition is 0 W/(m∙K), except for a deposition thickness of 0 mm. The function is not defined at this point because 0 mm deposit thickness with 0 W/(m∙K) thermal conductivity cannot be interpreted. When the thermal conductivity of the deposit is equal to that of the mold, there is no need to account for a decrease in heat conduction in the heat transfer calculations. It is sufficient to focus solely on the reduction in the diameter of the cooling channel. Because of the saturation character of cooling efficiency change as a function of deposit thickness, we chose to use sigmoid functions to describe the effect. A modified Hill’s sigmoid function (Eq. (17)) describes the process with the best fit (Fig. 4), which is widely used in biochemistry, physiology, and pharmacology:

17 $$\:{\eta\:}_{cooling}={\eta\:}_{cooling,\:max}\cdot \:\frac{{{k}_{deposit}}^{{C}_{1}}}{\left(1-\frac{{k}_{deposit}}{{k}_{mold}}\right)\cdot \:{{C}_{2}}^{{C}_{1}}+{{k}_{deposit}}^{{C}_{1}}}$$

where *η*_*cooling, max*_ (%) is cooling efficiency without deposition, *k*_*deposit*_ (W/(m∙K)) is the thermal conductivity of the deposition, *k*_*mold*_ (W/(m∙K)) is the thermal conductivity of the mold material, and *C*_*1*_ (-) and *C*_*2*_ (W/(mK)) are fitting parameters. *η*_*cooling, max*_ is known from simulation results carried out during the design process of the mold. *k*_*deposit*_ and *k*_*mold*_ can be approximated using literature and manufacturer data. C_1_ and C_2_ parameters must be fitted using fitting software. *η*_*cooling, max*_ decreases with increasing deposition thickness. However, it can be simplified without resulting in a significant error (3% error) by using the maximum cooling efficiency, which relates to the case where there is no deposition in the cooling system. We will use this simplification in this article.

**Fig. 5:**
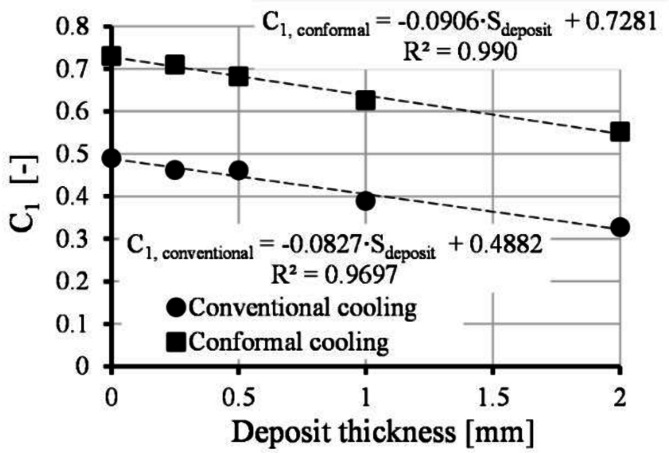
The value of fitting parameter C_1_ as a function of deposit thickness for conformal and conventional cooling systems.

The Origin, version 8.5 (OriginLab Corporation, Northampton, MA, USA) software was used for function fitting. We used the Levenberg-Marquardt algorithm to adjust the values of the parameters in the iterative procedure. The iteration aimed to minimize the chi-square sum. The fitting tolerance was set to 10^− 9^. After fitting the proposed model, we investigated the dependency of C_1_ parameter. Therefore, we plotted the C_1_ parameters as a function of deposition thickness. We observed that the C_1_ parameter decreases as a function of the deposition thickness shown in Fig. 5. The function can be described as a linear expression. Equation (18) expresses C_1_ parameter for conventional and conformal cooling systems:

18 $$\:{C}_{1}\left(CL,\:{S}_{deposit}\right)={C}_{3}\left(CL\right)\cdot \:{S}_{deposit}+{C}_{4}\left(CL\right),$$

where *S*_*deposit*_ (m) is deposit thickness, and *C*_*3*_ (1/m) and *C*_*4*_ (-) are fitting parameters. *C*_*3*_ and *C*_*4*_ values for both cooling systems can be seen in Fig. 5. Using Eq. (13), the C_1_ parameter can be calculated for both cooling systems; therefore, the C_1_ parameter must not be fitted.

**Fig. 6:**
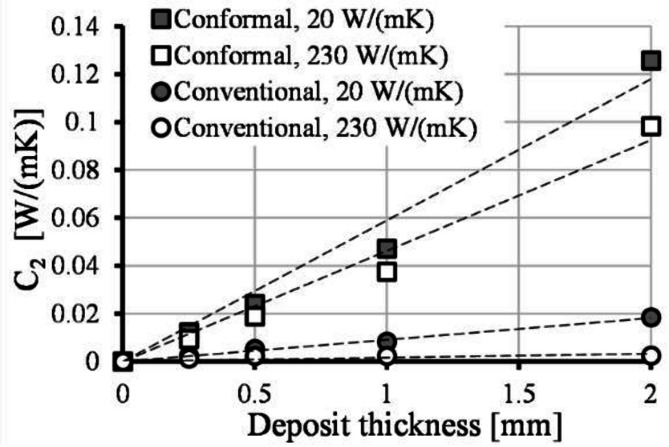
The value of fitting parameter C_2_ as a function of deposit thickness for different cooling layouts and mold thermal conductivities.

C_2_ values can also be expressed as a function of deposit thickness. C_2_ parameter shows a dependency on deposit thickness, cooling layout and thermal conductivity of the mold (Fig. 6). C_2_ parameter can be expressed using (Eq. (19)):

19 $$\:{C}_{2}\left(CL,\:{S}_{deposition},\:{k}_{mold}\right)={C}_{5}\left(CL,\:{k}_{mold}\right)\cdot \:{S}_{deposit}+{C}_{6}\left(CL,\:{k}_{mold}\right)$$

**Fig. 7:**
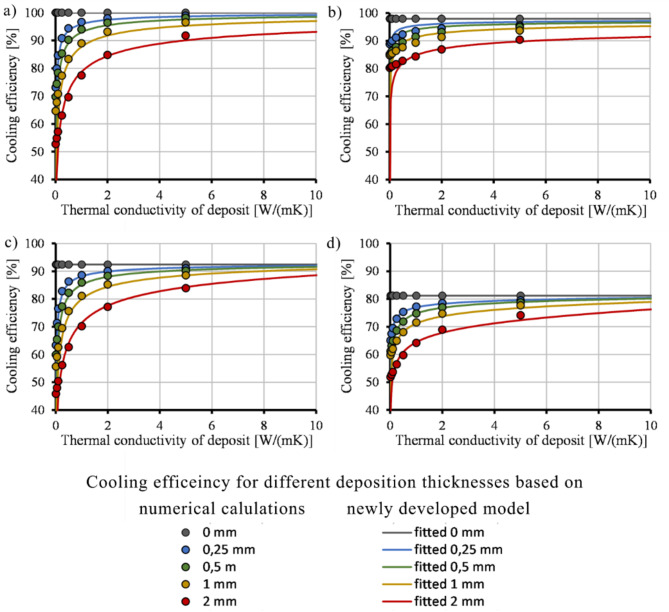
Cooling efficiency of the conformal AMP (**a**), conventional AMP (**b**), conformal MS1 (**c**) and conventional MS1 (**d**) inserts as a function of deposit thickness calculated based on the recalculated values of C_1_.

Using Eqs. (18) and (19), we calculated the cooling efficiency for each cooling layout (Fig. 7) with no significant difference in the goodness of fit compared to the first fitting. MAPE values were also similar to those of the original fitting. Hence, Eq. (18) and Eq. (19) can be used to simplify Eq. (17). *η*_*cooling, max*_ is calculated by numerical calculations that are carried out during the design process. *k*_*deposit*_ and *k*_*mold*_ can be approximated using literature and manufacturer data. Therefore, the cooling efficiency can be calculated for conformal and conventional cooling systems without fitting parameters. The following limits apply to the model:

- *0 (*%) ≤ *η*_*cooling, max*_ ≤ 100 (%),
- 0 (W/(mK)) ≤ k_deposit_ ≤ k_mold_ (W/(mK)),
- 0 (W/(mK)) < k_mold_ ≤ 230 W/(mK),
- 0 (mm) ≤ s_deposit_ ≤ R_cooling_ (mm).

For further parameters, the general injection molding parameter limits apply.

**Fig. 8:**
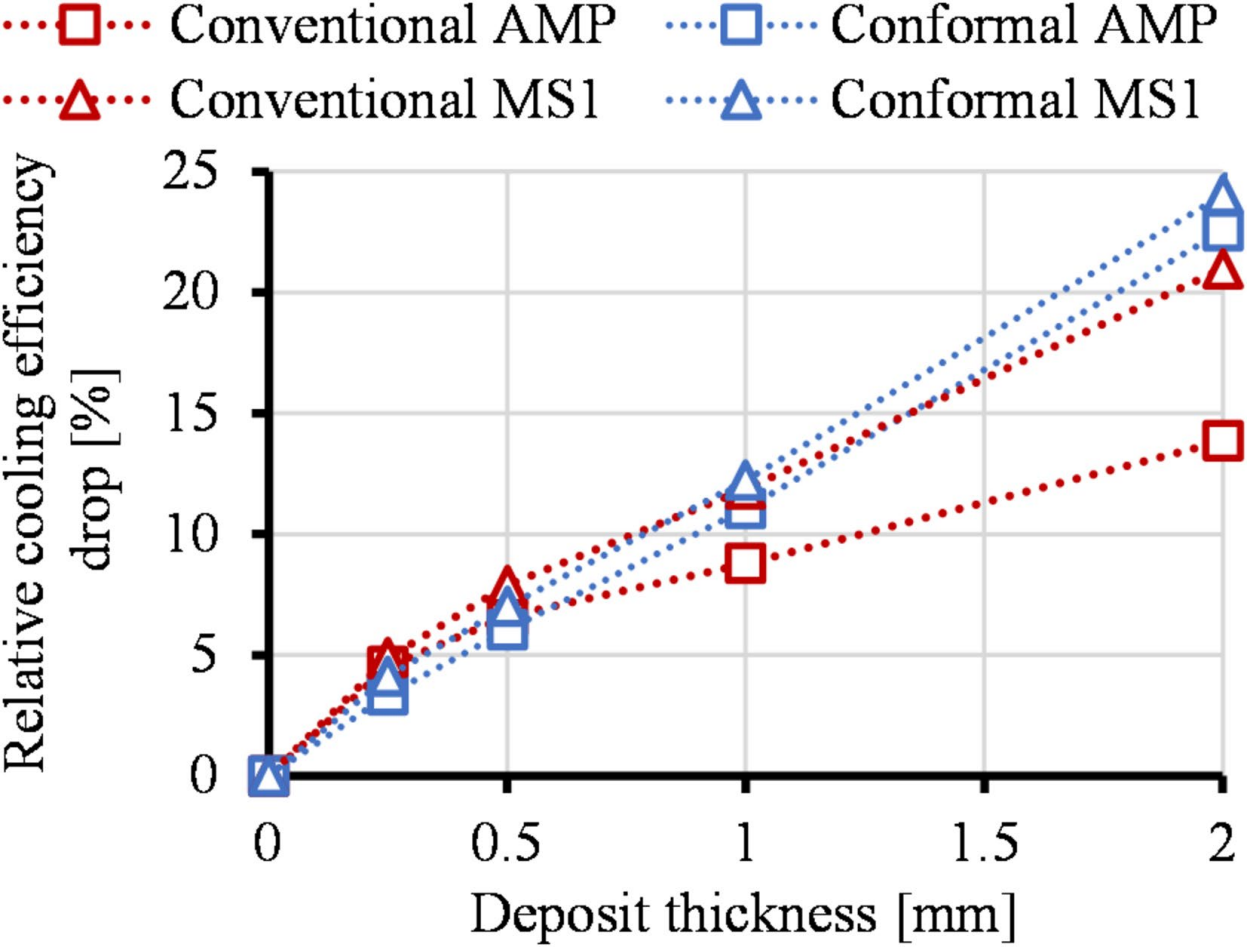
The relative cooling efficiency drop of each mold design as a function of deposit thickness.

We calculated the relative cooling efficiency drop using the results of the deposit of 1 W/(mK) thermal conductivity (Fig. 8.). The relative cooling efficiency drop can be expressed with Eq. (20):

20 $$\:{\eta\:}_{drop}=\frac{{\dot{Q}}_{channel,\:no\:deposition}-{\dot{Q}}_{\:channel,{s}_{dep}}}{{\dot{Q}}_{channel,\:no\:deposition}}\cdot \:100$$

where $\:{\dot{Q}}_{channel,\:no\:deposition}$ is the heat energy removed by the cooling channels without deposition for each mold design (*Conventional AMP*,* Conventional MS1 inserts*,* Conformal AMP and Conformal MS1)*, $\:{\dot{Q}}_{\:channel,{s}_{dep}}$ is the heat energy removed by the cooling channels with 0.25; 0.5; 1 and 2 mm deposit thickness with k = 1 W/(mK) deposit thermal conductivity for each mold design. The results show that conformal cooling channels are more sensitive to increases in deposit thickness than conventional channels. Furthermore the higher conductivity of the mold material mitigates the cooling efficiency drop caused by increasing deposition thickness. This effect is more prominent in the case of conventional cooling layouts.

## Conclusion

This study developed and validated a universal model for assessing the impact of deposition formed in the cooling channels on the cooling efficiency of injection mold inserts. We demonstrated that even modest deposition layers can substantially impede heat extraction by analyzing conventional and conformal cooling layouts manufactured from MS1 steel and high-conductivity copper-based Ampcoloy materials. These two materials represent the two extremes of thermal conductivity for high-series injection molds. Deposition layers that can form in the cooling channels during the use of the mold were modeled with various thicknesses. These depositions, like limescale or rust, have a low thermal conductivity compared to the mold material. Therefore, they hinder heat extraction and lower the cooling efficiency of the cooling channels. Numerical calculations were performed on the models with deposition thermal conductivities between 0.01 and 230 W/(m K) and deposit thicknesses of 0.25, 0.5, 1, and 2 mm. In the case of conformal cooling layouts, cooling efficiency decreased more with increasing deposit thickness than in the case of conventional solutions. This can lead to a low, conventional layout-like heat removal using a highly efficient conformal cooling layout, even at a deposit thickness of 1 mm. Using higher thermal conductivity material mitigates this effect. Relative cooling efficiency was calculated for various cases based on the heat removed through the cooling channels relative to the maximal heat removal of the best solution (*Conformal AMP* insert). The robust fitting parameters derived from our numerical simulations enable accurate predictions of cooling efficiency based on deposit thickness and thermal properties. We determined the fitting parameters for all insert types; therefore, cooling efficiency can be calculated for other mold geometries, mold materials, deposit thermal conductivities, and deposit thicknesses. These findings offer practical insights for predictive maintenance strategies, ensuring enhanced performance and longevity of injection molds.

## Data Availability

The data that support the findings of this study are available from the corresponding author upon reasonable request.

## Abbreviations

∆j: jth triangular integral element, –
∆T: Temperature difference, °C
a: Thermal diffusivity m^2^ s^−1^
c: Specific heat, J kg^−1^ K^−1^
C_1_: Fitting parameter, –
C_2_: Fitting parameter, W (mK)^−1^
C_3_: Fitting parameter, m^−1^
C_4_: Fitting parameter, –
C_5_: Fitting parameter, W (m^2^K)^−1^
C_6_: Fitting parameter, W (mK)^−1^
CL: Cooling layout, –
g: Gravitational acceleration, ms^−2^
h_coolant-deposition_: Heat transfer coefficient between coolant and deposition, Wm^−2^K^−1^
h_mold-air_: Heat transfer coefficient between mold and air, Wm^−2^K^−1^
h_mold-coolant_: Heat transfer coefficient between mold and coolant, Wm^−2^K^−1^
h_mold-deposition_: Heat transfer coefficient between mold and deposition, Wm^−2^K^−1^
i: Node number, –
j: Element number, –
k: Positioning parameter of the Hill function
*k*_*deposit*_: Thermal conductivity of the deposition, Wm^−1^K^−1^
k_e_: Effective thermal conductivity, W(mK)^−1^
*k*_*mold*_: Thermal conductivity of the mold material, Wm^−1^K^−1^
L: Length of the deposition, m
n: Parameter influencing the increase of the Hill function
q: Heat flux density, Wm^−2^
q*: Heat flux density in the normal direction, Wm^−2^
$\:\overline{\mathbf{q}}$: Cycle-averaged heat flux density, Wm^−2^
$\:{\dot{\mathbf{Q}}}_{\mathbf{c}\mathbf{h}\mathbf{a}\mathbf{n}\mathbf{n}\mathbf{e}\mathbf{l},\:\mathbf{m}\mathbf{a}\mathbf{x}}$: Maximum heat removal by the cooling channels, J
$\:{\dot{\mathbf{Q}}}_{\mathbf{c}\mathbf{h}\mathbf{a}\mathbf{n}\mathbf{n}\mathbf{e}\mathbf{l}}$: Heat removed by the cooling channels, J
R: Thermal resistance, KW^-1^
r_1_: Inner diameter of the deposition, m
r_2_: Outer diameter of the deposition, m
R_cooling_: Cooling channel radius, m
R_h_: Thermal resistance of convection, KW^−1^
R_λ_: Thermal resistance of conduction, KW^−1^
s: Local coordinate in the part thickness direction, m
*s*_*deposit*_: Deposit thickness, mm
t: Time, s
*T**: Fundamental solution of Laplace’s equation, K
*T*_*a*_: Air temperature, K
*T*_*c*_: Coolant temperature, K
v: Velocity, ms^−1^
y_max_: Saturation level of Hill function
*η*_*cooling*_: Relative cooling efficiency, %
η_cooling, max_: Cooling efficiency without deposition, %
ρ: Density, kgm^−3^
